# An MPS-BNS Mixed Strategy Based on Game Theory for Wireless Mesh Networks

**DOI:** 10.1155/2013/936536

**Published:** 2013-01-17

**Authors:** S. Q. Huang, G. C. Wang, H. H. Zhen, Z. Zhang

**Affiliations:** ^1^Network and Education Technology Center, Jinan University, Guangzhou, Guangdong 510632, China; ^2^School of Computer and Electrical Information, Guangxi University, Nanning, Guangxi 530004, China; ^3^College of Information Science and Technology, Jinan University, Guangzhou, Guangdong 510632, China

## Abstract

To achieve a valid effect of wireless mesh networks against selfish nodes and selfish behaviors in the packets forwarding, an approach named mixed MPS-BNS strategy is proposed in this paper. The proposed strategy is based on the Maximum Payoff Strategy (MPS) and the Best Neighbor Strategy (BNS). In this strategy, every node plays a packet forwarding game with its neighbors and records the total payoff of the game. After one round of play, each player chooses the MPS or BNS strategy for certain probabilities and updates the strategy accordingly. In MPS strategy, each node chooses a strategy that will get the maximum payoff according to its neighbor's strategy. In BNS strategy, each node follows the strategy of its neighbor with the maximum total payoff and then enters the next round of play. The simulation analysis has shown that MPS-BNS strategy is able to evolve to the maximum expected level of average payoff with faster speed than the pure BNS strategy, especially in the packets forwarding beginning with a low cooperation level. It is concluded that MPS-BNS strategy is effective in fighting against selfishness in different levels and can achieve a preferable performance.

## 1. Introduction

In wireless networks such as ad hoc and mesh networks, the steady operation of the networks must rely on the cooperation for which packets should be forwarded sufficiently between nodes, namely, the routers in networks. However, for the sake of saving battery life for their own communications, some nodes in wireless network do not cooperate to the basic network functioning, making the network lack trust between nodes. These nodes refuse to forward packets by dropping other's packets and trying to use others to send their own. This noncooperative behavior is named selfish behavior, and these routers are selfish nodes. Users or nodes that want to maximize their own welfare and do not contribute to the network are defined as selfish nodes or free riders [1]. Now the selfish behavior is an important issue and is being vigorously researched. The simulation study presented in Michiardi and Molva [2] showed that the performance of multihop wireless networks severely degrades in face of selfish node's misbehavior. To deal with this problem, many mechanisms have been proposed to detect the selfish nodes and restrict their selfish behaviors in wireless networks. In terms of the previous work on this problem, there are main approaches based on the credit approach, the reputation approach, network coding approach, game theory, and so on.

In the credit approach [3–7], cooperation is induced by means of payments received every occasion when a node acts as a relay and forwards a packet, and such credit can later be used by these nodes to encourage others to cooperate.

Reputation-based schemes observe the behavior of their neighboring nodes through promiscuous overhearing and accordingly assign them a reputation rating which is used for identifying the selfish nodes. In reputation-based schemes [8–14], a node's behavior is measured by its neighbors using a watchdog mechanism, and nodes can be punished for noncooperation. To deal with the issue, cooperative nodes sometimes are perceived as being selfish because of unreliable transmission. Joang et al. [15] proposed a method based on subjective logic to discover the trust networks between specific parties. Kane and Browne [16] successfully transplanted and applied subjective logic to a wireless network environment. Due to the unreliability and lack of information of the wireless networks, the pure subjective logic-based model may lead to a high uncertainty value in some cases. To solve the problem, a reputation computation model was proposed to discover and prevent selfish behaviors by combining familiarity values with subjective opinions [17]. And to compute the reputation of the nodes in wireless networks, some techniques such as network coding and fuzzy recommendation were proposed [18, 19].

For the credit approach and reputation approach, because the system needs a central control requiring an infrastructure, this method cannot be used in the distributed environment. Game theory can easily cope with selfish behaviors and therefore was introduced to the research of selfish nodes related to wireless mesh networks. To adapt with the distributed environment, some detecting methods based on game theory were proposed in recent studies [20–25].

A distinct and novel approach named best neighbor strategy (BNS) was proposed to stimulate and enforce cooperation among such selfish nodes in an ad hoc or wireless mesh network environment, where there is no central authority to monitor their unacceptable behaviors [26]. Komathy and Narayanasamy [26] pointed out that there were four basic methods using game theory to defend selfish nodes in an ad hoc network. This paper is to discuss the first and third ones. The first is to choose the best response as a rational expectation according to the expected behavior of the other players, and this method is called maximum payoff strategy (MPS) in this paper. The third one is to choose the other player's strategy, if it is achieving more than others. Based on the third method, Komathy and Narayanasamy proposed the BNS [26] against selfish neighbors. In BNS, each node records the total payoff of its every neighbor in each round of the packet forwarding game. Once one round is completed, the player changes its current strategy to its neighbor's current strategy, if the neighbor has achieved a higher total return than any other neighbor. BNS is able to converge faster to enforce cooperation among selfish nodes and is robust against selfishness when invaded by selfish behaviors.

In our paper, beginning with high cooperation level, BNS is able to provide a superior performance; however, it displays inferior behaviors beginning with low cooperation level, which will be discussed in the simulation sections in this paper. To improve the performance of BNS in low cooperation level, we then combine MPS and BNS to propose a scheme identified as mixed MPS-BNS strategy against selfish nodes and selfish behaviors. In this paper, the basic and complete models of mixed MPS-BNS strategy are firstly discussed and analyzed, respectively, with two repeated packet forwarding (RPF) games of two player and multiplayer. Then, via the simulation of the RPF games, the performance of the true BNS and the mixed MPS-BNS will be compared and discussed on average payoff and cooperation level.

## 2. MPS-BNS Strategic Game of Two Player

### 2.1. The Rules of the Game

In this section, the restriction of the mixed MPS-BNS strategy on selfish behaviors is illustrated by a simple two-player game. In the game, neighboring nodes p and −p are playing a repeated packet forwarding game G, which consists of *n* generations, and each generation consists of *L* rounds of play. Each node eventually chooses one of the strategies, Forward (F) or Drop (D).

In MPS-BNS strategy, each node has a strategy space *S*, consisting of two policies, namely, MPS-BNS and ALL Drop. The strategy space of a player is shown in Table 1, in which each player always drops others' packets in a certain probability of *α*(0 ≤ *α* ≤ 1) and chooses MPS-BNS strategy in a probability of (1 − *α*). The probability *α* represents the selfishness level and also simulates the mutation proportion. Each player chooses MPS and BNS strategies in probabilities of *β*(1 − *α*)(0 ≤ *β* ≤ 1) and (1 − *α*)(1 − *β*), respectively. MPS and BNS have two substrategies Forward (F) and Drop (D) as shown in Table 2, which also shows the payoff matrix. In MPS strategy, the player chooses the strategy (F or D) with which they will get the maximum payoff in the next round according to its neighboring player. In BNS strategy, the player chooses the strategy (F or D) with which its neighboring player gets the maximum payoff in the previous round.

According to different prime strategies, there exits four states in Markov process as follows.

- State (1,1): both player 1 and player 2 have MPS-BNS as their prime strategies for the current game.
- State (1,2): player 1 has MPS-BNS as its prime strategy, and player 2 has ALL-D as its prime-strategy for the current game.
- State (2,1): player 1 has ALL-D as its prime strategy for the current game, and player 2 has MPS-BNS as its prime strategy.
- State (2,2): both player 1 and player 2 have ALL-D as their prime strategy for the current game.

The expected payoff for player 1 in state (1,1) is simulated using Table 3 with parameters *R*_*i*_, *S*_*i*_, *P* and *T*, which represent different payoffs gained by the nodes in different strategies. In MPS strategy, every node chooses the strategy which will get the maximum payoff corresponding to the strategies of its neighbors; while in BNS strategy, every node follows the strategy of its neighbor having the maximum payoff in previous round. Accordingly, the payoff of MPS is higher than that of BNS; thus, *R*_1_ = 5.5, *R*_2_ = 5, *R*_3_ = 4.5, *R*_4_ = 4 and *S*_1_ = 3.5, *S*_2_ = 3, *P* = 1, *T* = 1. The *α*_1_ and *α*_2_ are the proportions of ALL-D chosen by player 1 and player 2, respectively.

### 2.2. The Expected Payoff

Let ep_1_^*i*^ be the expected payoff of player 1 in the *i*th state and can be computed as

(1) ep11=(1−α1){β[(1−α2)β(L·R1)  +(1−α2)(1−β)(L·R2)+α2(S1·L)] +(1−β)[(1−α2)β(L·R3)+(1−α2)     ×(1−β)(L·R4)+α2(S2·L)]}+α1[(1−α2)(T·L)+α2(L·P)],ep12=(1−α1){β[α2β(L·R1)  +α2(1−β)(L·R2)+(1−α2)(S1·L)] +(1−β)[α2β(L·R3)+α2(1−β)     ×(L·R4)+(1−α2)(S2·L)]}+α1[(1−α2)(P·L)+α2(L·T)],ep13=α1{β[(1−α2)β(L·R1)  +(1−α2)(1−β)(L·R2)+α2(S1·L)] +(1−β)[(1−α2)β(L·R3)+(1−α2)(1−β)     ×(L·R4)+α2(S2·L)]}+(1−α1)[(1−α2)(T·L)+α2(L·P)],ep14=α1{β[α2β(L·R1)+α2(1−β)(L·R2)  +(1−α2)(S1·L)]+(1−β) ×[α2β(L·R3)  +α2(1−β)(L·R4)+(1−α2)(S2·L)]}+(1−α1)[(1−α2)(P·L)+α2(L·T)].

Similarly, the expected payoff ep_2_^*i*^ of play 2 in state *i* can be computed. The expected average payoffs for player 1 and player 2 at equilibrium state are derived as [26]

(2) ep1eq=(1L)[abcd]·(ep11 ep12 ep13 ep14),ep2eq=(1L)[abcd]·(ep21 ep22 ep23 ep24),

where *a*, *b*, *c*, and *d* are the convergence probabilities of the Markoff process of the game at state (1,1), state (1,2), state (2,1), and state (2,2) at equilibrium state [26], respectively. Thus, the expected payoff per player is

(3) epeq=ep1eq+ep2eq2.

**Scheme 1:**
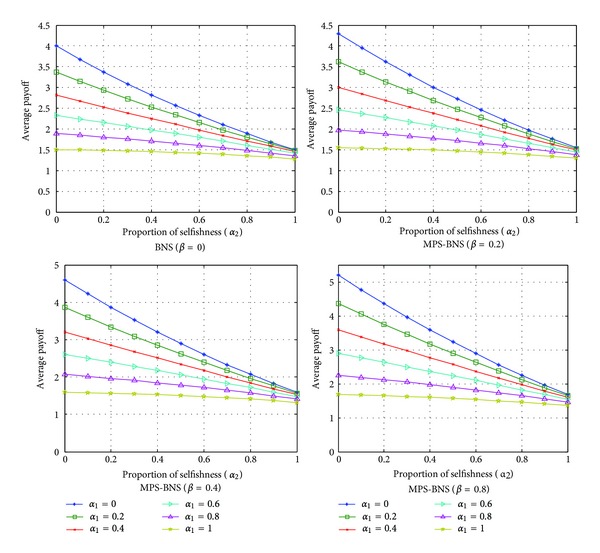
Expected average payoff per player against selfishness.

Scheme 1 illustrates the expected payoff per player calculated by (3) against noncooperative players comparing BNS and MPS-BNS. The average payoff per player gradually decreases as the selfishness of each player *α*_1_ and *α*_2_ increases. When selfishness is zero (*α*_1_ = *α*_2_ = 0), BNS (*β* = 0) gives both players a share of 4, which is the maximum payoff, while MPS-BNS (*β* > 0) gives higher maximum payoff as the proportion of MPS *β* increases. With the same selfishness of each player *α*_1_ and *α*_2_ in other value, MPS-BNS also provides a higher average payoff than BNS.

## 3. MPS-BNS Strategic Game of Multiplayer

### 3.1. Evolution with MPS-BNS as Pure Strategy

The pure strategy means that every node in network has a uniform strategy profile (Forward or Drop) against all its neighbors. When MPS-BNS is implemented as pure strategy, as shown in Table 4, each node in topology has MPS and BNS strategies with the probability of *β*(0 ≤ *β* ≤ 1) and (1 − *β*), respectively, against all its neighbors *n*_*k*_(*k* = 1,2,…, *m*), where *k* is the number of neighbors.

**Scheme 2:**
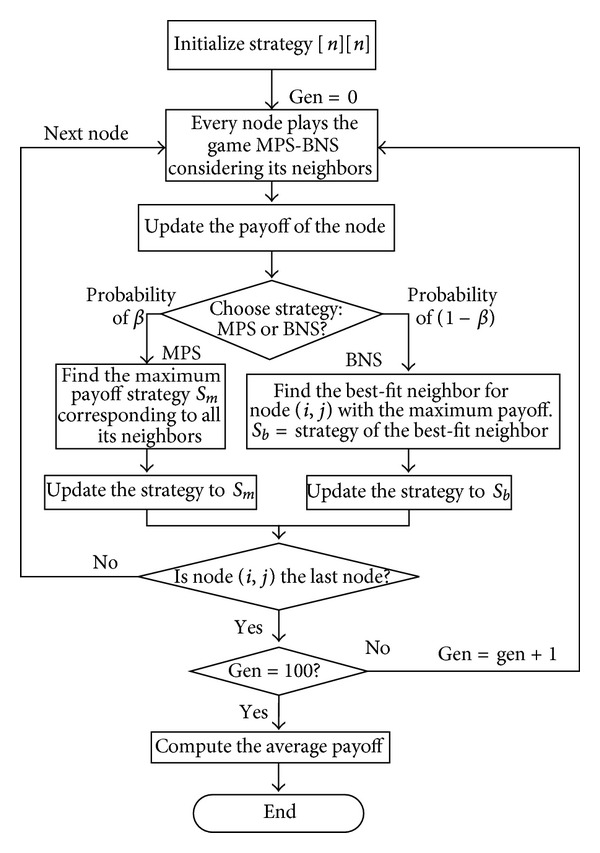
The game of MPS-BNS as pure strategy.

Scheme 2 is a flow chart to illustrate the strategic game of multiplayer using MPS-BNS as a pure strategy. The computing method of average payoff per player in Scheme 1 is given as follows:

(4) average  payoff=Pn×n,

where *P* is the total payoff of all nodes, and *n* × *n* is the size of topology.

**Scheme 3:**
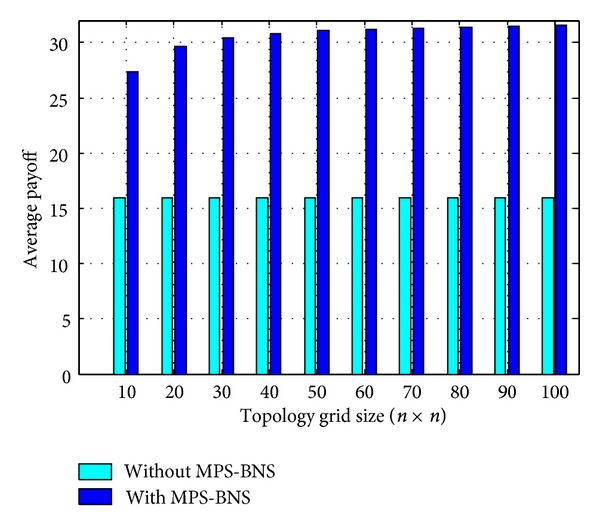
Average payoffs per player of evolution of MPS-BNS as pure strategy.

The proposed model MPS-BNS is implemented using tool MATLAB. The topology size ranges from 10 × 10 to 100 × 100, and the simulation runs for 100 generations. The simulation results portrayed in Scheme 3 are the average values from running the simulation for about 20 times with random initial strategies and probability of choosing MPS *β* (0 ≤ *β* ≤ 1). In the strategy without MPS-BNS, all nodes have random strategies Forward or Drop, choosing each strategy with probability of 0.5. In Scheme 3, the outcome of MPS-BNS as pure strategy is much higher than that without MPS-BNS and is able to evolve to the maximum expected level at about 32. The average payoff of the strategy without MPS-BNS remains the same at 16 in all topology grid sizes. When the size of topology increases to 50 × 50, the evolution speed slows down, and the maximum average payoff per player remains at the maximum expected level.

### 3.2. Evolution with MPS-BNS as Mixed Strategy

**Scheme 4:**
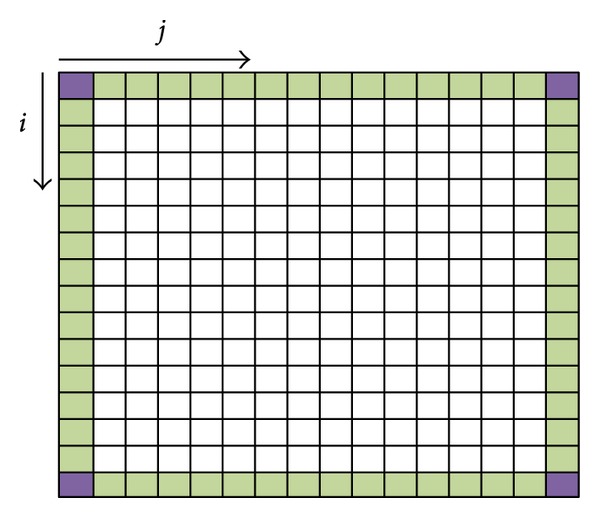
*n* × *n* network grid.

Instead of having a uniform strategy profile in pure strategy, in mixed strategy, every node in topology maintains a strategy profile with different strategies against neighbors.  Scheme 4 depicts a size of *n* × *n* topology grid, in which the corner node has three neighbors, the edge node (excluding corner node) has five neighbors, and each of the other nodes has eight neighbors. Every node in grid maintains a strategy profile, *S*_(*i*,*j*)_ = {*s*_1_, *s*_2_,…, *s*_*k*_,…, *s*_*m*_} as shown in Table 5, where (*i*, *j*) is the coordinate of node, *s*_*k*_ is the strategy adapted against neighbor node *k*, and *m* is the number of neighbors.

**Scheme 5:**
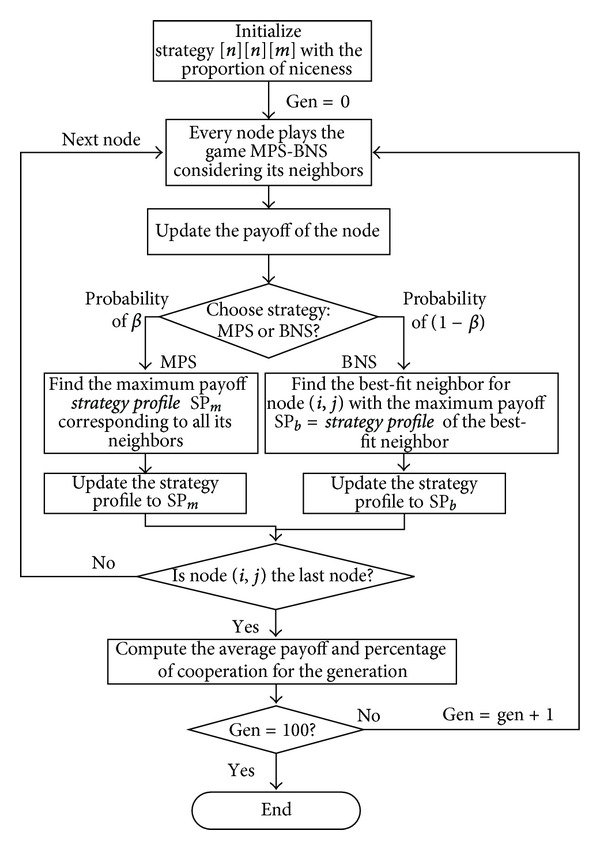
The game of MPS-BNS as mixed strategy.

The level of generosity named niceness representing the proportion of a node beginning with cooperation is introduced, and the performance metrics are explored.  Scheme 5 illustrates the algorithm to play a game of MPS-BNS as a mixed strategy profile. The percentage of cooperation in Scheme 5 is computed as follows:

percentage cooperation = (*m*/(*n* × *n*)) × 100%, *m* is the number of nodes having forwarding strategy.

**Scheme 6:**
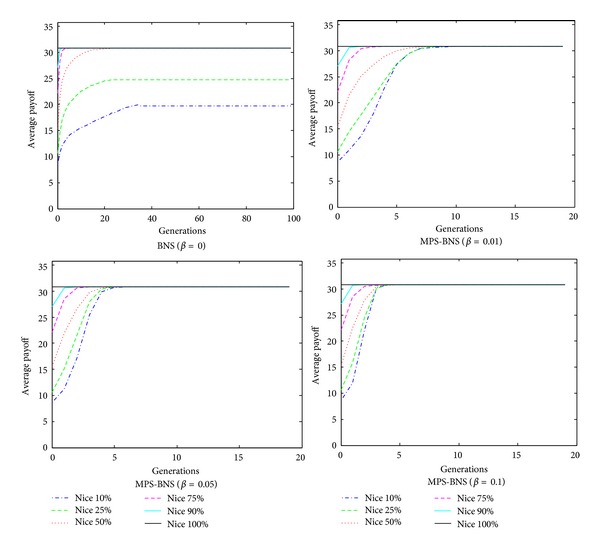
The average payoff of evolution of MPS-BNS with mixed strategy profile.

**Scheme 7:**
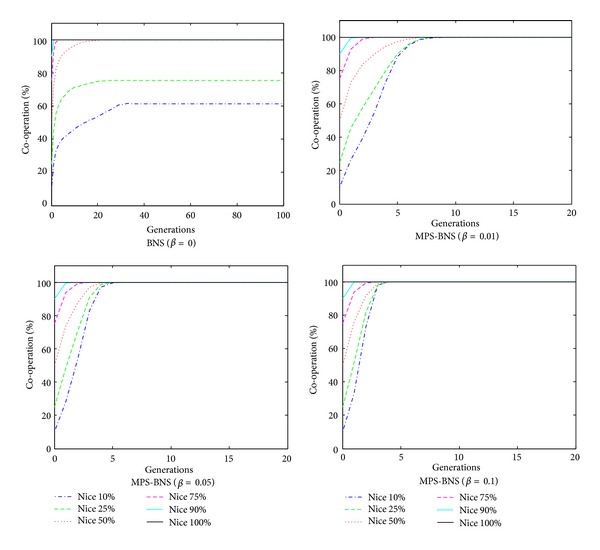
The Percentage of cooperation of evolution of MPS-BNS with mixed strategy profile.

The evolution of game of MPS-BNS is simulated by MATLAB. The game evolves for 100 generations in the 40 × 40 topology. The probability (*β*) of choosing MPS ranges from 0.01 to 0.1. The strategies of evolution are initialized with different proportions of niceness: 0.1, 0.25, 0.5, 0.75, 0.9, and 1.0. The probability of a node being benign is proportional to the proportion of niceness introduced. Schemes 6 and 7 portray the average payoff and percentage of cooperation in the evolution of MPS-BNS with mixed strategy profile in different niceness and probability choosing MPS. In Scheme 6, when *β* = 0, representing the true BNS strategy, BNS behaves advanced performance of evolving to the maximum level in fast convergence speed with niceness more than 70%; however, as niceness decreases, the convergence slows down; thus with niceness less than 25%, the outcomes are unable to evolve to the maximum level but a lower average payoff instead. The mixed MPS-BNS (*β* > 0) behaves better performance than the true BNS in low niceness. When *β* ≥ 0.01, representing the proportion of MPS to be equal to or greater than 0.01 in mixed MPS-BNS, in all proportion of niceness, all the outcomes of MPS are able to converge to the maximum average payoff 31, and as *β* increases, the convergence converses faster, especially when *β* = 0.1 convergences are all within the first five generations in all proportion of niceness. The evolution of percentage of cooperation in Scheme 7 is similarly the same as the average payoff in Scheme 6. As the proportion of MPS (*β*) increases, the network experiences better cooperation from nodes and converges faster. From the simulation, we find that though the probability of choosing MPS *β*(0 ≤ *β* ≤ 1) is just slightly more than zero and much less than the probability of choosing BNS, the outcomes of MPS-BNS behave much better than the true BNS especially with low level of niceness, that is because in MPS the maximum payoff strategy is chosen in a more direct way than BNS, bringing a much faster convergence speed.

### 3.3. MPS-BNS against Selfishness

**Scheme 8:**
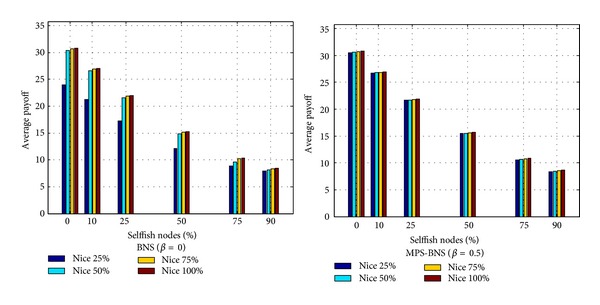
Average payoff per player of MPS-BNS with niceness against selfish nodes.

**Scheme 9:**
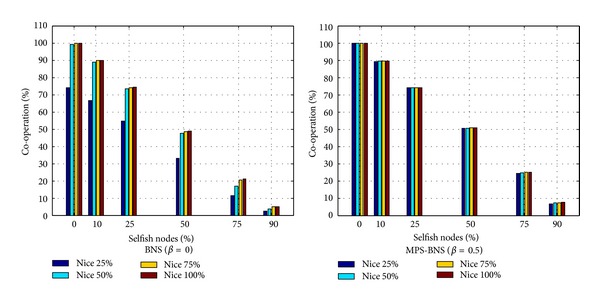
Level of cooperation of MPS-BNS with niceness against selfish nodes.

In this section, the behavior of the topology with the induction of selfish nodes is examined under a mixed strategy profile. In the topology with selfish nodes, a proportion of nodes always behaves noncooperatively and drops packets straightway without choosing MPS-BNS in the evolution. Schemes 8 and 9 depict the average payoff per player and the level of cooperation of MPS-BNS against various proportions of selfish nodes. The topology contains 40 × 40 as the population size and evolves for 100 generations. As the percentage of selfish nodes increases, the average payoff and level of cooperation decrease. The true BNS strategy (*β* = 0) can evolve to a high level of average payoff and cooperation at all levels of selfishness with niceness ≥ 50%; however, it behaves much lower performance with niceness of 25% than with niceness ≥ 50%, especially at a low level of selfishness. The mixed MPS-BNS (*β* = 0.5, representing the choice of MPS and BNS with the same probability) behaves approximately the same high level of performance in various proportions of niceness. MPS-BNS especially with the niceness of 25% gives relatively a distinctly better share of average payoff at all levels of selfishness. In addition, MPS-BNS with various proportions of niceness achieves higher average payoff and cooperation than BNS with 75% and 90% percentage of selfish nodes. Schemes 8 and 9 conclude that MPS-BNS has better networking performance and better robustness than BNS in fighting against selfishness.

## 4. Conclusion

The paper has proposed a mixed MPS-BNS strategy based on game theory against selfish nodes and selfish behaviors in the process of packets forwarding in wireless mesh networks. Through our research, the true BNS is able to provide a superior network performance with high initial cooperation levels but behaves inferior with low initial cooperation levels. To overcome this problem, we combine BNS with MPS strategy, each of which is chosen with respective probability. In MPS strategy, every node selects the strategy which will get the maximum payoff corresponding to the strategies of its neighbors. In BNS strategy, every node follows the strategy of its neighbor having the maximum payoff. A basic MPS-BNS strategic game of two players is discussed and is extended to a complicated strategic game involving multiplayer. The simulation and discussions of the proposed strategy as pure strategy and mixed strategy are carried out on performance of average payoff and cooperation level. The results conclude that MPS-BNS is able to converge to the expected maximum level with various proportions of the initial percentage of cooperation and converge more rapidly than BNS. The simulation results of MPS-BNS against selfishness conclude that MPS-BNS behaves superior robustness than BNS defending against selfish nodes. Thus, the proposed MPS-BNS strategy is much more effective and efficient in defending against selfishness in wireless networks.

## Figures and Tables

**Table 1:** Strategy space.

MPS-BNS (1 − *α*)				ALL-D (*α*)
MPS (1 − *α*)*β*(1 − *α*)		BNS (1 − *α*)(1 − *β*)		Drop (D)
Forward (F)	Drop (D)	Forward (F)	Drop (D)	

**Table 2:** Sub strategies.

Player 1	Player 2	
	F	D
F	4, 4	3, 0
D	0, 3	1, 1

**Table 3:** Payoff matrix of player 1 in the state (1,1).

Player 1	Player 2		
	MPS-BNS (1 − *α*_2_)		ALL-D (*α*_2_)
	MPS (1 − *α*_2_)*β*	BNS (1 − *α*_2_)∗(1 − *β*)
MPS-BNS (1 − *α*_1_)			
MPS (1 − *α*_1_)*β*	$\begin{array}{c} \left(1-\alpha_{2}\right)\beta *\left(L\cdot R_{1}\right) \end{array}$	$\begin{array}{c} \left(1-\alpha_{2}\right)\left(1-\beta \right)*\left(L\cdot R_{2}\right) \end{array}$	*α*_2_(*S*_1_ · *L*)
BNS (1 − *α*_1_)(1 − *β*)	$\begin{array}{c} \left(1-\alpha_{2}\right)\beta *\left(L\cdot R_{3}\right) \end{array}$	$\begin{array}{c} \left(1-\alpha_{2}\right)\left(1-\beta \right)*\left(L\cdot R_{4}\right) \end{array}$	*α*_2_(*S*_2_ · *L*)
ALL-D (*α*_1_)	(1 − *α*_2_)(*T* · *L*)		*α*_2_(*L* · *P*)

**Table 4:** Uniform strategy profile of MPS-BNS as pure strategy.

Probability	*n*_1_	*n*_2_	*⋯*	*n*_*k*_	*⋯*	*n*_*m*_
*β*	MPS (F or D)					
(1 − *β*)	BNS (F or D)					

**Table 5:** The strategy profile with MPS-BNS as mixed strategy.

Probability	*s*_1_	*s*_2_	*⋯*	*s*_*k*_	*⋯*	*s*_*m*_
*β*	MPS_1_	MPS_2_	*⋯*	MPS_*k*_	*⋯*	MPS_*m*_
(1 − *β*)	BNS_1_	BNS_2_	*⋯*	BNS_*k*_	*⋯*	BNS_*m*_
